# Perceived Pain during Rapid Maxillary Expansion (RME): Trends, Anatomical Distinctions, and Age and Gender Correlations

**DOI:** 10.1155/2021/7396466

**Published:** 2021-07-14

**Authors:** Emanuela Serritella, Stefania Migliaccio, Ludovica Musone, Alessandra Impellizzeri, Adriana Assunta De Stefano, Gabriella Galluccio

**Affiliations:** ^1^Department of Oral and Maxillofacial Sciences, “Sapienza” University of Rome, Rome, Italy; ^2^Department of Orthodontics, Faculty of Dentistry, Central University of Venezuela, Caracas, Venezuela

## Abstract

**Objectives:**

To investigate pain trends and characteristics of different facial districts in patients undergoing rapid maxillary expansion (RME) and its possible correlations with age and gender.

**Materials and Methods:**

85 subjects (45 males and 40 females) undergoing RME were selected and analyzed during first two weeks of treatment. Patients rated daily two types of pain perception: the general perceived pain (GPP), i.e., the pain overall perceived in the face, and the local perceived pain (LPP), i.e., the pain perceived locally in the following anatomical areas: anterior palate (APA), posterior palate (PPA), nasal (NA), joint (JA), and zygomatic (ZA). Patients were provided the Numeric Rating Scale (NRS) and Wong–Baker Faces Pain Rating Scale (FPS) to correctly assess their GPP and LPP. Pearson correlation coefficient and analysis of variance (ANOVA) were, respectively, used to define the linear relationship between all the variables considered and to verify whether the response variables (gender and age) were significantly different (*α* < 0.05).

**Results:**

Sample's mean age was 10.11 years. Average pain values of GPP and LPP progressively rise from day 1 to days 2-3 (pain peak) and tended to decrease until day 14, with a linear decrease for GPP and a not linear decrease for LPP. PPA and APA resulted the most painful areas, followed, respectively, by JA, ZA, and NA. Statistically significant differences resulted in average pain values according to patients' age and gender, both in GPP and LPP.

**Conclusion:**

RME causes perception of pain in several maxillofacial areas. Pain reported during RME resulted positively correlated with age and gender of patients.

## 1. Introduction

Fear of suffering is often a major deterrent against beginning an orthodontic treatment and is the primary cause of discontinuity and lack of compliance in patients undergoing long-term procedures [1]. Pain and discomfort occur during all types of orthodontic procedures, such as separator placement, archwire placement and activations, application of orthopedic forces, and debonding. Patients wearing fixed appliances reported higher values for intensities of pressure, tension, pain, and teeth sensitivity when compared with patients wearing removable appliances [2]. The greatest levels of discomfort and pain were reported by patients undergoing fixed orthodontic therapies and orthopedic therapies [1, 2]. The active phase of palatal expansion is variable in length, generally lasting 10–14 days, with patients reporting pain mainly during the first days of device activation [1, 2]. Despite the importance of this factor in clinical practice, orthodontic pain is rarely scientifically investigated, especially with regards to common fixed orthodontic therapies, such as rapid maxillary expansion (RME) [3, 4]. Variable amounts of orthopedic force are generated in RME of median palatal sutures. This force is absorbed and propagated in the three planes of the craniofacial complex through tissue displacement and remodeling mechanisms that exert pressure on the bones surrounding the maxilla via cranial and circummaxillary sutures [3, 4]. Analyzing stress and force distribution during RME on craniofacial structures, Jafari et al. have observed a high level of stress dissemination to all circummaxillary sutures and important bone displacements, not only in the anterior and posterior palate but also in nasal and zygomatic bones [5]. Moreover, with respect to growing age, it has been demonstrated that RME is capable of modifying the condyle-fossa relationship and of affecting the joint area [6].

Several clinical studies have investigated pain related to RME, mostly comparing the use of different activation protocols (2 turns/die vs. 1 turn/die) or of different types of appliances [7, 8]. However, there has been little study of the perception of pain in craniofacial districts other than those that are most heavily loaded, i.e., the palate and upper teeth. The single study (Önçağ et al.) that has examined RME-related pain perception in 5 craniofacial areas (palatal, dental, malar, frontal, and temporal) reported increased pain perception in the dental and palatal areas compared to the others and a significant statistical difference in average pain for all the anatomical districts considered [9].

The aim of this study is to analyze pain trends and characteristics and the possible correlations with age and gender variables, during the first 14 days of RME therapy, observing patient perceived pain not only in the palatal area but also in the nasal, joint, and zygomatic areas.

## 2. Materials and Methods

A consecutive series of patients under the age of 14 undergoing RME therapy in the Orthodontics Department of the Sapienza University Hospital of Rome were asked to participate in the study, from March 1^st^ to December 27^th^ 2019, a total of 96 patients. The contraction of the maxillary arch and the presence of a mono or bilateral cross-bite were criteria for inclusion. Intellectual disability, metabolic/chronic disease, current use of pain medication, previous orthodontic treatment, or failure to give informed consent by each patient's parents were criteria for exclusion. This study was approved by the Institutional Ethics Committee (N.53/18–0000711), and informed consent was obtained from each patient's parents.

All patients underwent expansion therapy of the upper jaw using a rapid palatal expander (RPE) that was attached to bands on the first maxillary molars with traditional hyrax screws (A0620 SS, manufactured by Leone S.p.A, Florence, Italy). The RPE appliance activation protocol, which lasted 14 days, required 2 activations per day: 1 in the morning and 1 in the evening.

The participants were asked to avoid analgesic medication throughout the activation period; those who took medication of this type during the period of therapy were later excluded from the study.

At the time of positioning of the palate expansion appliance, parents were instructed about the methods and activation times of the appliance. All patients received a pain assessment card and were instructed how to correctly fill out the form, which was then returned once completed. Participants were asked to indicate their pain perception at the end of each day, precisely 30–60 minutes after the second daily activation, for all 14 days of treatment. To minimize participant dropout, patient's parents were asked to set an alarm clock and check the proper compilation of the pain assessment card every day.

Both of the scientifically recognized scales for pain assessment [10, 11], the Numeric Rating Scale (NRS) and the Wong–Baker Faces Pain Rating Scale (FPS), were used; the former was used to evaluate the general pain perceived during the day and the latter to evaluate pain perceived in specific anatomical areas.

**Figure 1:**
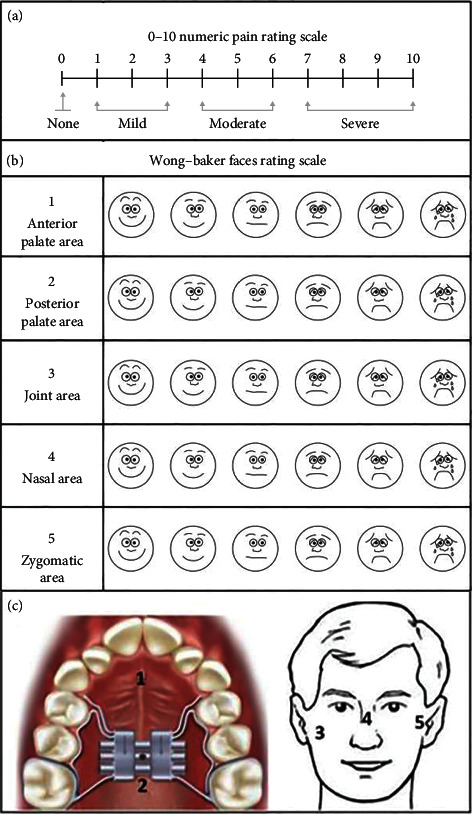
Pain scales used for daily evaluation. (a) NRS. (b) Wong–Baker FPS. (c) Picture used to facilitate patients' identification of anatomical areas.

General perceived pain (GPP): overall perceived pain during the day. The pain self-assessment scale used was the Numeric Rating Scale (NRS) (Figure 1(a)).

Local perceived pain (LPP): pain perceived during the day related to a specific anatomical zone. The areas considered were the anterior palate area (APA), posterior palate area (PPA), joint area (JA), nasal area (NA), and zygomatic area (ZA) (Figure 1(b)).

The areas were also represented graphically on the card with numbers to facilitate the evaluation (Figure 1(c)). The pain self-assessment scale used was the Wong–Baker Faces Pain Rating Scale (FPS).

### 2.1. Statistical Analysis

All data obtained were examined using SAS software (version 9.4). Statistical analysis identified several different indicators (mean, median, standard deviation, max and min), which were used to construct a line plot graph to represent the distribution. The Shapiro–Wilk test was used to test the normality assumption of data. A Pearson correlation coefficient was used to define the linear relationship between all the variables considered. Analysis of variance (ANOVA) was used to verify whether the response variables (gender and age) were significantly different. The threshold for statistical significance was set at *α* < 0.05.

## 3. Results

A total of 96 patients participated in the study. However, 7 subjects were excluded because of incomplete data, and 4 subjects were excluded because they took pain medication during treatment. Thus, the final number of study participants was 85 patients: 45 males and 40 females. The age range was 7–14 years, with a median age of 10.11 years (Table 1).

All patients (100%, *n* = 85) reported general pain (NRS) during the 14 days of the study and in all the anatomical areas examined (FPS). The mean pain range for GPP was from 2.58 (day 14) to 6.17 (day 2), using the NRS scale. The mean pain range for LPP was from 0.23 (ZA_day 11) to 4.82 (PPA_day 2), using the FPS scale.

### 3.1. General Perceived Pain (GPP)

**Figure 2:**
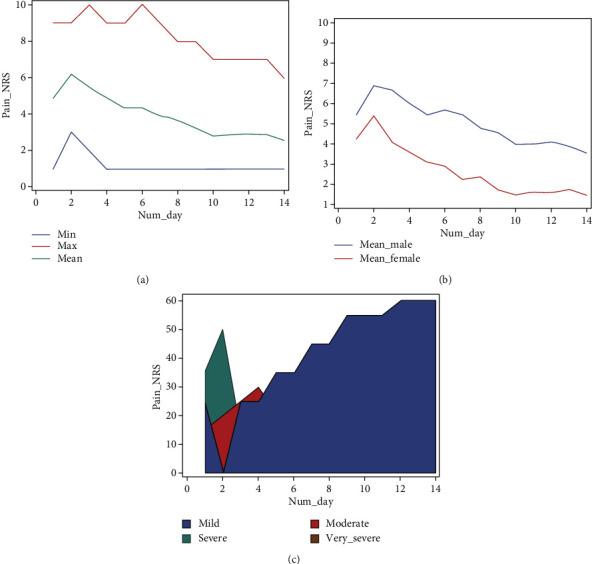
Pain related to RME in the first 2 weeks of treatment according to the NRS scale. (a) Pain values over time in all patients and (b) in male and female patients. (c) Qualitative perception over time in all patients.

Figure 2 shows the trend and quality of perceived pain, according to the NRS scale. Males reported higher average pain values (5.02_NRS) than females (2.58_NRS) for each day of treatment (Figure 2(b)). On day 2, the highest pain values were reported by both male (6.89_NRS) and female (5.37_NRS) patients.

An age-related analysis reveals differences in pain perception between all ages under investigation. Results for each age group are listed in the decreasing order of average pain values during the 14 days of study (NRS): “13 y” (average = 5.57; 5 Pt.), “12 y” (average = 5.28; 15 Pt.), “11 y” (average = 4.96; 10 Pt.), “14 y” (average = 4.57; 5 Pt.), “10 y” (average = 4.05; 15 Pt.), “9 y” (average = 3.31; 15 Pt.), “7 y” (average = 2.46; 10 Pt.), and “8 y” (average = 1.96; 10 Pt).

The ANOVA *t*-test supports “gender” and “age” as statistically significant variables (*α* < 0.05).

### 3.2. Local Perceived Pain (LPP)

**Figure 3:**
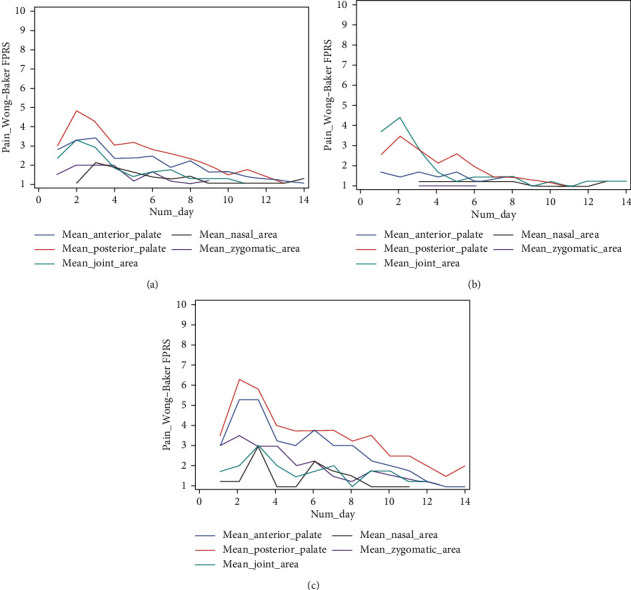
Pain related to RME in the first 2 weeks of treatment according to the Wong–Baker Scale in the different districts analyzed (a) and according to gender: male (b) and female (c).

Figure 3 shows the pain trend in each analyzed area, according to the FPS scale. All the averages by the area are listed in Table 1. Females reported higher pain values than males for every considered area except for JA (Table 1, Figures 3(b) and 3(c)). On days 2 and 3, the highest pain values were reported by both male and female patients, differently according to the anatomical area analyzed. Day 2 resulted the pain peak day for the areas APA (F = 5.25_FPS/M = 1.55_FPS) and PPA (F = 6.25_FPS/M = 3.55_FPS). Day 3 resulted the pain peak day for the areas NA (F = 3.00_FPS/M = 1.33_FPS) and ZA (F = 3.00_FPS/M = 1.11_FPS). Concerning the area JA, day 3 resulted the pain peak day for females (3.00_FPS) and day 2 for males (4.44_FPS).

There were differences in the pain perception of patients of different ages in each of the areas analyzed. Results are listed in Table 2 in a decreasing order, from the age reporting the most pain to the one reporting the least pain during the 14 days of therapy.

The pain trend was not linear across the areas examined, so the “Pearson correlation coefficient” was applied to evaluate whether any linear correlation existed among the different variables. In terms of pain increase, positive linear correlations were found among peak days and several of the following days (*ρ* > 0.7). In particular, there was a strong relationship of dependence among peak days 2 and 3 and days 6 and 8, for all investigated anatomical districts (0.72 < *ρ* < 0.94) (Table 3). It, therefore, was decided to examine these four days more closely. The results of this analysis are listed in Table 4 under Supplementary Materials Section, organized according to gender and age.

The ANOVA *t*-test demonstrated significant differences between the “gender” and “age” variables (*α* < 0.05), except for in JA, where gender was not significant.

Supplementary data related to the ANOVA test analysis of both GPP and LPP are listed in Table 5 under Supplementary Materials Section.

## 4. Discussion

Clinical studies have demonstrated pain related to RME as a frequent symptom, reported by 66, 12–99% of patients [12, 13]. These data are confirmed by the present study, in which 100% of subjects undergoing RME (*n* = 85) reported pain throughout the entire active phase of therapy (NRS) and in all the examined anatomical districts (FPS).

Analysis of general perceived pain (GPP) indicates pain was greatest during the first 6 days of activation, with a maximum peak at day 2 (NRS: 6, 18) and tended to decrease gradually in the following days; these findings concur with the current literature [7, 13, 14]. However, the quality of RME-related pain reported in our study is not consistent with those of previous studies. Indeed, though GPP pain levels were mostly described as mild throughout the treatment period, they were referred to as moderate or strong by the majority of our participants during the first days of activation. Needleman et al. [7] also reported high pain levels, especially after the first 6 screw turns; during this period, 69% of patients, moreover, had to take pain medication. Geçgelen Cesur and Aksoy [12] indicated moderate pain levels during the initial 7 days of therapy. Two other studies demonstrate pain presence throughout the entire therapy, but with very low reported values [14, 15].

However, our local perceived pain (LPP) analysis resulted in average pain levels inferior to the NRS, with FPS ranging from 1.10 to 2.51. Even painful days (days 2, 3, 6, and 8) resulted in mild discomfort according to this analysis; furthermore, we saw great variability between the anatomical districts examined. These outcomes, together with the conflicting evidence in the existing literature, draw attention to the difficulties surrounding subjective pain evaluation even using validated scales as well as to the necessity of further investigating how other variables (gender, age, psychological factors, and hormonal factors) contribute to pain evaluation and extreme individual variability.

In this regard, interesting gender-related and age-related results were found by this study, including a statistically significant difference between male and female pain perception. While males reported higher pain values than females for GPP (NRS), this evidence was contradicted by their reporting of LPP (FPS). Females, in fact, reported higher LPP (FPS) pain values in all considered facial districts, except the joint area (JA), which is also the only area showing no statistical significance.

Though several clinical studies of RME-related pain have not identified significant gender differences [7, 8, 13], others similarly demonstrate females experiencing significantly more pain than males [14, 15]. Variability in pain perception based on sex and gender has been long debated. Genetic, molecular, physiological, and psychosocial factors contribute to differences in processing pain and pain perception in men and women. In particular, women's threshold for pain is greater, more varied, and more variable than for men.[16]. In a study including children and adolescents, Allen et al. [17] noticed important sex differences in the cortisol-pain relationship. Increase in cortisol was positively associated with greater pain tolerance in males and greater pain sensitivity in females. A literature review by Berkley et al. [16] highlighted the importance of gender in pain perception and inflammation, underlining the influence of hormonal modulation on nociception through factors such as estradiol, menstrual cycle, or the sex-related effects of NSAIDs and ASICs. These findings validate the existence of a gender-related difference in pain perception during RME, though increased sensitivity in females only occurred in LPP.

Our age-related analysis also pointed to significant variations in evaluations of both GPP and LPP. Though studies of pain and its correlation with age and aging show increased perception of discomfort with age, research on the prevalence of pain in children and adolescents displays inconsistent findings, and it is difficult to reach general conclusions concerning pain prevalence and characteristics in this particular population group [18]. Haraldstad et al. [19] reported that pain increases with age, with girls between 16 and 18 reporting the highest discomfort. A study by Blankenburg et al. [20], of perception of different nociceptive stimuli, including pressor and mechanical stimulation, found that children are more sensitive to most painful stimuli than adolescents and also noted that growth-related changes during puberty seem to influence pain perception. At the craniomaxillofacial level, these different pain perceptions may be explained by tissue and morphological differences in bones structures related to age changes. During craniofacial growth, sutures represent secondary growth centers that respond to mechanical stress with various structural effects: sutural interdigitation becomes more complex with increase in age. Median palatal sutures respond to RME with a greater expansion rate at the age of 8 than in patients who are 12, 13, or 14 years old [2]. In this study, GPP results support the evidence in the literature, with greater reported pain as age increases: patients aged 7-8 reported inferior pain values than older patients; the values reported by patients aged 12-13 were especially high. However, our LPP results show great variability among examined districts as well as highest pain values in patients aged 8 (mean = 2.66) and 14 (mean = 2.14). The lowest values were reported by patients aged 11 (mean = 1.09) and 13 (mean = 1.26).

The differences emerging from comparison of the two analyses may be due to the use of different scales, NRS and FPS, and reflect the findings of previous studies that have also used both [10, 11].

There were also interesting trends our LPP findings on pain location and timing. As expected, the posterior and anterior palate areas resulted in the highest pain values. It is interesting to note that the nasal area, the closest anatomical area and the one experiencing the greatest changes after RME, was the district in which the lowest average pain level was reported. However, some pain was reported for every examined district. Jafari et al. observed the deep anatomical effects of RME appliances, reporting the highest stress levels in the areas of the maxillary bone, zygomatic process, external walls of the orbit, frontozygomatic suture, and the frontal process of maxilla [5]. Interestingly, these areas of high-stress distribution coincide with some of the most painful anatomical districts of this study. These findings are suggestive of the role of circummaxillary sutures in modulating orthodontic pain perception, as a constraint on the transmission of the expansion forces to the other neighbouring anatomical districts.

As with the GPP findings, using the NRS scale, reported LPP pain was greater in the first day of the activation of the appliance, unlike the GPP findings; however, there is no clear linearity in the decrease of LPP pain over time. Various increases in pain values, different for each examined area, were noticed from day 3 to day 14. The pain values reported on days 6 and 8, in particular, were strongly correlated with the peak days, in all the areas considered (*ρ* > 0.7). Some studies on cranial sutures undergoing mechanical stress could explain this pain “reactivation” over time. Cleall et al. [21] reported the presence of highly vascularized connective tissue with moderate chronical inflammation response inside the sutural bone of monkeys undergoing RME after 14 days of treatment. Investigating histological changes in the mean palatine suture in patients undergoing RME, Caprioglio et al. [22] later reported the presence of a highly vascularized and coagulum-rich central osteoid matrix, especially on day 7 of activation. A recent murine study by Wu et al. [23] describes a particular arrangement and orientation of new bone formation in expanding sutures, with the largest volumetric increase on day 7 of expansion. Finally, an interesting investigation by Che et al. [24] on the role of the nonneural cholinergic system in bone remodeling after RME shows increasing values of ACh and an increasing RANK/OPG ratio after 1, 3, and 7 days of expansion. The presence of pronounced bone remodeling phenomena, such as ACh, seems to align with the results about pain development obtained in our study, which indicate days 6 and 8 as the most related to average pain peak days (days 2 and 3). These inflammation processes involve increased molecular expression that we know to be involved in pain modulation.

Despite the interesting results obtained, this study presents some limitations. The patient sample examined is too limited to represent reliable results regarding the characteristics of RME-related pain, especially in connection with patients' age and gender. Furthermore, the pain assessment was limited to patient self-assessment, but the importance of using multiple methods of pain assessment, given the complexity of changes that this symptom can undergo during experimental procedures, especially in a children's population, needs to be emphasized.

## 5. Conclusions

- RME therapy caused pain in the entire study population at the palate, joint, zygomatic, and nasal areas
- Age and gender were positively correlated with overall pain perception and with pain perception in every single area analyzed except the joint area
- In all examined facial areas, perceived pain trends do not decrease linearly; further studies are needed to deeply analyze if bone remodeling and inflammation processes during RME might modulate pain perception over time.

## Figures and Tables

**Table 1:** Basic characteristics of the participants and average pain values (FPS) in the different anatomical areas analyzed in the first 2 weeks of treatment.

Characteristics	Subjects		
	Male	Female	Total
Age, years, mean (SD)	10.89 (1.93)	9.25 (1.73)	10.11 (2.00)
Gender, number (%)	45 (53%)	40 (47%)	85 (100%)

Pain values (FPS), mean (SD)			
Anterior palate area (APA)	1.41 (0.27)	2.75 (1.39)	2.04 (0.73)
Posterior palate area (PPA)	1.70 (0.98)	3.43 (1.34)	2.51 (1.11)
Joint area (JA)	1.67 (1.22)	1.64 (0.54)	1.65 (0.82)
Nasal area (NA)	1.30 (0.32)	1.67 (0.66)	1.10 (0.37)
Zygomatic area (ZA)	0.94 (0.33)	1.29 (1.05)	1.19 (0.57)

**Table 2:** Average pain values (FPS) in the different anatomical areas analyzed in the first 2 weeks of treatment, according to patient age.

Anterior palate (APA)		Posterior palate (PPA)		Joint area (JA)		Nasal area (NA)		Zygomatic area (ZA)	
Average pain^*∗*^	Patient age	Average pain^*∗*^	Patient age	Average pain^*∗*^	Patient age	Average pain^*∗*^	Patient age	Average pain^*∗*^	Patient age
3.14 ± 1.70	13	4.36 ± 2.06	8	4.57 ± 1.55	8	4.00 ± 2.22	14	3.68 ± 2.08	14
2.78 ± 1.76	8	3.57 ± 1.70	7	2.48 ± 1.42	9	1.48 ± 0.98	10	1.82 ± 1.31	9
2.64 ± 0.84	7	3.14 ± 1.87	13	1.78 ± 0.58	7	1.44 ± 1.01	11	1.48 ± 1.01	8
2.52 ± 1.69	10	2.48 ± 1.58	10	1.71 ± 1.25	12	1.09 ± 0.49	12	1.33 ± 0.83	10
2.14 ± 0.99	12	2.48 ± 1.53	12	1.14 ± 1.87	14	1.00 ± 1.27	9	1.18 ± 0.66	12
1.21 ± 0.89	11	1.78 ± 0.80	11	0.71 ± 1.81	11	0.43 ± 0.75	7	0.30 ± 0.75	11
1.14 ± 0.89	9	1.43 ± 2.11	9	0.095 ± 0.24	10	0.14 ± 0.53	8	0.01 ± 0.53	7
0.86 ± 1.87	14	1.00 ± 2.04	14	0.00 ± 0.00	13	0.00 ± 0.00	13	0.00 ± 0.00	13

^*∗*^Mean ± standard deviation.

**Table 3:** Linear correlations among peak days 2 and 3 and days 6 and 8, for all investigated anatomical districts, according to Pearson correlation coefficient.

—	*ρ* ^*∗*^	*P* value^∗∗^
Anterior palate (APA)		
Day 2_day 6	0.73	<0.0001
Day 2_day 8	0.72	<0.0001
Day 3_day 6	0.83	<0.0001
Day 3_day 8	0.79	<0.0001

Posterior palate (PPA)		
Day 2_day 6	0.74	<0.0001
Day 2_day 8	0.85	<0.0001
Day 3_day 6	0.79	<0.0001
Day 3_day 8	0.72	<0.0001

Joint area (JA)		
Day 2_day 6	0.73	<0.0001
Day 2_day 8	0.77	<0.0001
Day 3_day 6	0.83	<0.0001
Day 3_day 8	0.78	<0.0001

Nasal area (NA)		
Day 2_day 6	0.72	<0.0001
Day 2_day 8	0.74	<0.0001
Day 3_day 6	0.94	<0.0001
Day 3_day 8	0.76	<0.0001

Zygomatic area (ZA)		
Day 2_day 6	0.85	<0.0001
Day 2_day 8	0.76	<0.0001
Day 3_day 6	0.83	<0.0001
Day 3_day 8	0.81	<0.0001

^*∗*^ *ρ*, Pearson correlation coefficient; positive linear correlation for 0.72 < *ρ* < 0.94. ^∗∗^*P* value <0.0001.

## Data Availability

The data used to support the findings of this study are included within the article and the datasets are available from the corresponding author upon request.

## Supplementary Materials

Supplementary Materials Table 4: Pain levels in the different anatomical areas analyzed on days 2, 3, 6, and 8 (FPS). ^*∗*^Mean ± standard deviation. Table 5: ANOVA procedure supplementary data of NRS and FPS scales analysis, according to age and gender response variables.
