# Supplementary material for: Perceived Pain during Rapid Maxillary Expansion (RME): Trends, Anatomical Distinctions, and Age and Gender Correlations
Source: Pain Res Manag. 2021 Jul 14;2021:7396466. doi: 10.1155/2021/7396466 (PMC8295004; doi:10.1155/2021/7396466)
Supplement: Supplementary Materials — Table 4: Pain levels in the different anatomical areas analyzed on days 2, 3, 6, and 8 (FPS). ∗Mean ± standard deviation. Table 5: ANOVA procedure supplementary data of NRS and FPS scales analysis, according to age and gender response variables. [file 7396466.f1.docx]

**Table 4.** Pain levels in the different anatomical areas analyzed on days 2, 3, 6 and 8 (FPS).

*Mean ± st.deviation

|  | PATIENTS | | | AGE | | | | | | | |
| --- | --- | --- | --- | --- | --- | --- | --- | --- | --- | --- | --- |
| VARIABLE | **TOT *** | **FEMALE*** | **MALE*** | **7*** | **8*** | **9*** | **10*** | **11*** | **12*** | **13*** | **14*** |
| DAY_2_APA | 3.29 ± 3.16 | 5.25 ± 3.03 | 1.55 ± 2.08 | 4.00 ± 4.22 | 5.00 ± 5.27 | 2.67 ± 2.58 | 4.67 ± 1.95 | 2.00 ± 2.11 | 2.67 ± 2.58 | 4.00 ± 0 | 0 |
| DAY_3_APA | 3.41 ± 3.70 | 5.25 ± 3.91 | 1.78 ± 2.60 | 4.00 ± 4.22 | 5.00 ± 5.27 | 2.00 ± 1.69 | 5.33 ± 4.25 | 3.00 ± 3.16 | 3.33 ± 3.52 | 2.00 ± 0 | 0 |
| DAY_6_APA | 2.47 ± 3.05 | 3.75 ± 3.42 | 1.33 ± 2.13 | 2.00 ± 2.11 | 5.00 ± 5.27 | 0 | 4.67 ± 2.58 | 1.00 ± 1.05 | 2.67 ± 2.58 | 0 | 4.00 ± 0 |
| DAY_8_APA | 2.23 ± 2.57 | 3.00 ± 2.86 | 1.55 ± 2.08 | 2.00 ± 2.11 | 3.00 ± 3.16 | 0.67 ± 0.97 | 4.00 ± 3.38 | 1.00 ± 1.05 | 2.00 ± 2.93 | 4.00 ± 0 | 2.00 ± 0 |
| DAY_2_PPA | 4.82 ± 2.85 | 6.25 ± 1.88 | 3.55 ± 2.98 | 8.00 ± 2.11 | 4.00 ± 4.22 | 6.00 ± 1.69 | 4.00 ± 1.69 | 2.00 ± 2.11 | 6.00 ± 0 | 6.00 ± 0 | 0 |
| DAY_3_PPA | 4.23 ± 4.13 | 5.75 ± 4.23 | 2.89 ± 3.58 | 5.00 ± 5.27 | 7.00 ± 3.16 | 2.00 ± 2.93 | 5.33 ± 4.25 | 4.00 ± 4.22 | 4.00 ± 4.47 | 6.00 ± 0 | 0 |
| DAY_6_PPA | 2.82 ± 2.93 | 3.75 ± 3.27 | 2.00 ± 2.33 | 3.00 ± 3.16 | 7.00 ± 3.16 | 0 | 3.33 ± 2.58 | 1.00 ± 1.05 | 2.67 ± 2.58 | 4.00 ± 0 | 4.00 ± 0 |
| DAY_8_PPA | 2.35 ± 2.60 | 3.25 ± 2.85 | 1.55 ± 2.08 | 3.00 ± 3.16 | 3.00 ± 1.05 | 0 | 4.00 ± 3.38 | 2.00 ± 2.11 | 2.00 ± 2.93 | 2.00 ± 0 | 4.00 ± 0 |
| DAY_2_JA | 3.29 ± 3.65 | 2.00 ± 3.36 | 4.44 ± 3.54 | 2.00 ± 2.11 | 8.00 ± 2.11 | 4.67 ± 3.52 | 0 | 5.00 ± 5.27 | 2.67 ± 2.58 | 0 | 4.00 ± 0 |
| DAY_3_JA | 2.94 ± 3.24 | 3.00 ± 3.65 | 2.89 ± 2.88 | 3.00 ± 3.16 | 7.00 ± 3.16 | 3.33 ± 2.58 | 0.67 ± 0.97 | 0 | 4.67 ± 3.52 | 0 | 4.00 ± 0 |
| DAY_6_JA | 1.65 ± 2.86 | 1.75 ± 3.42 | 1.55 ± 2.29 | 2.00 ± 2.11 | 5.00 ± 5.27 | 2.67 ± 1.95 | 0 | 0 | 2.00 ± 2.93 | 0 | 0 |
| DAY_8_JA | 1.29 ± 2.06 | 1.00 ± 1.75 | 1.55 ± 2.29 | 2.00 ± 2.11 | 2.00 ± 2.11 | 2.67 ± 1.95 | 0 | 0 | 2.00 ± 2.93 | 0 | 0 |
| DAY_2_NA | 1.06 ± 1.84 | 1.25 ± 1.73 | 0.89 ± 1.93 | 0 | 0 | 0 | 1.33 ± 0.97 | 2.00 ± 2.11 | 1.33 ± 1.95 | 0 | 6.00 ± 0 |
| DAY_3_NA | 2.12 ± 3.27 | 3.00 ± 3.65 | 1.33 ± 2.70 | 0 | 0 | 3.33 ± 4.88 | 2.00 ± 1.69 | 3.00 ± 3.16 | 2.00 ± 2.93 | 0 | 8.00 ± 0 |
| DAY_6_NA | 1.41 ± 2.65 | 2.25 ± 3.42 | 0.67 ± 1.35 | 0 | 0 | 3.33 ± 4.88 | 0.67 ± 0.97 | 2.00 ± 2.11 | 1.33 ± 1.95 | 0 | 4.00 ± 0 |
| DAY_8_NA | 1.41 ± 2.04 | 1.50 ± 2.20 | 1.33 ± 1.91 | 0 | 2.00 ± 2.11 | 1.33 ± 1.95 | 1.33 ± 1.95 | 3.00 ± 3.16 | 0.67 ± 0.97 | 0 | 4.00 ± 0 |
| DAY_2_ZA | 2.00 ± 3.00 | 3.50 ± 3.61 | 0.67 ± 1.35 | 0 | 2.00 ± 2.11 | 3.33 ± 4.88 | 2.67 ± 0.97 | 0 | 2.67 ± 3.90 | 0 | 4.00 ± 0 |
| DAY_3_ZA | 2.00 ± 3.30 | 3.00 ± 3.79 | 1.11 ± 2.54 | 0 | 1.00 ± 1.05 | 3.33 ± 4.88 | 2.67 ± 3.90 | 1.00 ± 1.05 | 1.33 ±1.95 | 0 | 8.00 ± 0 |
| DAY_6_ZA | 1.65 ± 2.78 | 2.25 ± 3.27 | 1.11 ± 2.15 | 0 | 1.00 ± 1.05 | 3.33 ± 4.88 | 2.00 ± 1.69 | 0 | 1.33 ± 1.95 | 0 | 6.00 ± 0 |
| DAY_8_ZA | 1.06 ± 1.56 | 1.25 ± 1.41 | 0.89 ± 1.68 | 0 | 2.00 ± 2.11 | 0.67 ± 0.96 | 2.00 ± 1.69 | 0 | 0.67 ± 0.97 | 0 | 4.00 ± 0 |

**Table 5.** Supplementary data of ANOVA procedure for NRS and FPS analysis, according to age and gender.

*DF= degrees of freedom; **Pr>F= confidence interval

|  | **Gender** | | | **Age** | | |
| --- | --- | --- | --- | --- | --- | --- |
|  | **DF*** | **F value** | **Pr>F**** | **DF** | **F value** | **Pr>F** |
| ***NRS*** | 1 | 428.22 | <.0001 | 7 | 63.51 | <.0001 |
| ***FPS*** |  |  |  |  |  |  |
| *APA* | 1 | 87.21 | <.0001 | 7 | 14.75 | <.0001 |
| *PPA* | 1 | 119.68 | <.0001 | 7 | 22.01 | <.0001 |
| *JA* | 1 | 0.03 | 0.8661 | 7 | 52.72 | <.0001 |
| *NA* | 1 | 9.74 | 0.0019 | 7 | 34.42 | <.0001 |
| *ZA* | 1 | 90.90 | <.0001 | 7 | 34.92 | <.0001 |
